# Successful treatment of a persistent air leak with an endobronchial valve in a 17‐year‐old patient with necrotizing pneumonia

**DOI:** 10.1002/rcr2.70053

**Published:** 2024-11-06

**Authors:** Nina M. Janssen, Rein Posthuma, Sophie Kienhorst, Michiel A. G. E. Bannier, Ulrich C. Lalji, Frits M. E. Franssen, Roy T. M. Sprooten

**Affiliations:** ^1^ Department of Respiratory Medicine Maastricht University Medical Center Maastricht The Netherlands; ^2^ Department of Pediatric Respiratory Medicine MosaKids Children's Hospital, Maastricht University Medical Center Maastricht The Netherlands; ^3^ Department of Radiology and Nuclear Medicine Maastricht University Medical Center Maastricht The Netherlands

**Keywords:** bronchopleural fistula, necrotizing pneumonia, persistent air leak, unidirectional endobronchial valve

## Abstract

Pleural empyema is a severe condition associated with high morbidity and mortality. Treatment usually consists of pleural drainage with chest tube or surgery, in combination with antimicrobial treatment. Severe pneumonia can evolve in a necrotizing pneumonia, given a higher susceptibility to the occurrence of bronchopleural fistulas with persistent air leaks. This complicates recovery, and surgery may not always be the optimal treatment. We present a case involving a 17‐year‐old female patient who experienced a post‐operative persistent air leak due to necrotizing pneumonia after video‐assisted thoracic surgery decortication for empyema, which was successfully treated using an endobronchial valve. After 6 months the valve was removed without complications. Follow‐up imaging and lung function revealed a limited area of atelectasis and minimal pleural thickening with normal lung volumes.

## Graphical abstract

We present a case involving a 17‐year‐old female patient who experienced a post‐operative persistent air leak due to necrotizing pneumonia after Video‐Assisted Thoracic Surgery decortication for empyema, which was successfully treated using an endobronchial valve. Follow‐up imaging and lung function revealed a limited area of atelectasis and minimal pleural thickening with normal lung volumes.

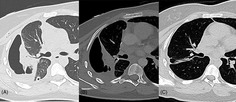

## INTRODUCTION

Necrotizing pneumonia, a severe complication of bacterial lung infections, can lead to bronchopleural fistula formation, resulting in a persistent air leak (PAL). Managing PALs is often challenging, with traditional treatments like chest tube drainage and surgery offering limited success. Endobronchial valves (EBV) have emerged as a promising alternative. This case report describes the successful resolution of a persistent air leak caused by necrotizing pneumonia and a bronchopleural fisula using EBV placement.

## CASE REPORT

**FIGURE 1:**
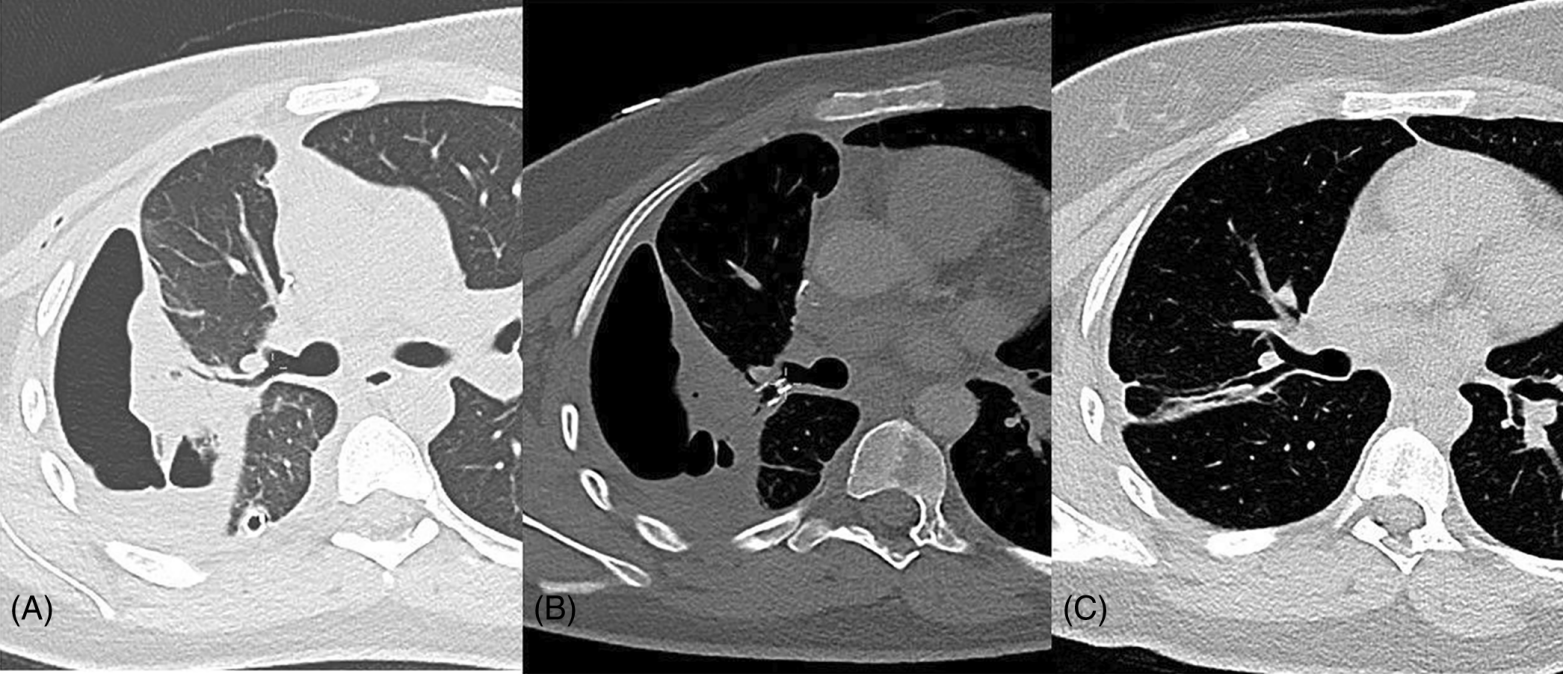
(A) Chest computed tomography (CT) scan showing necrotizing pneumoniae of the right upper lobe with a chest tube in place, before placing the endobronchial valve. (B) Chest CT scan showing a reduction of cavetating lesion of a necrotizing pneumonia of the right upper lobe 1 month after placement of endobronchial valve with the endobronchial valve in place. (C) Chest CT scan 1 year after placement en 6 months after the subsequent removal of the endobronchial valve, showing a limited area of atelectasis and minimal pleural thickening.

A 17‐year–old female patient, without any prior medical history, was referred to the our institution due to pleural empyema. She had been admitted at a local hospital 4 days earlier with dyspnea, hypoxia, fever and productive cough attributed to a consolidation in the dorsal segment of the right upper lobe. During her two‐day hospitalization, she received supplemental oxygen and oral antibiotic therapy (amoxicillin/clavulanic acid). This was switched after 1 day to amoxicillin following a positive pneumococcal antigen test. After 2 days, she was discharged with oral amoxicillin but was readmitted 2 days later due to pleural effusion. A chest tube was inserted, which drained 1200 mL of purulent fluid before she was referred to our tertiary center. A chest computed tomography scan (CT scan) revealed necrotizing lung infection with empyema (Figure 1A). There was a very high suspicion for the presence of a bronchopleural fistula, given the air configurations in the abscessing part of the lung, which seemed to be connected to air configurations reaching just outside the visceral pleura. Therefore combined intrapleural therapy with tissue plasminogen activator (tPA) and deoxyribonuclease (DNase) to synergistically enhance drainage was contra‐indicated. Instead, video‐assisted thoracic surgery (VATS) decortication was decided for. During surgery, a defect in the upper right lobe was observed, corresponding to the necrotizing area seen on the CT scan. Despite optimal treatment, a PAL of 350–550 mL/min was observed with hydropneumothorax 3 weeks postoperatively, using a digital thoracic monitoring and drain system (Thopaz).

**FIGURE 2:**
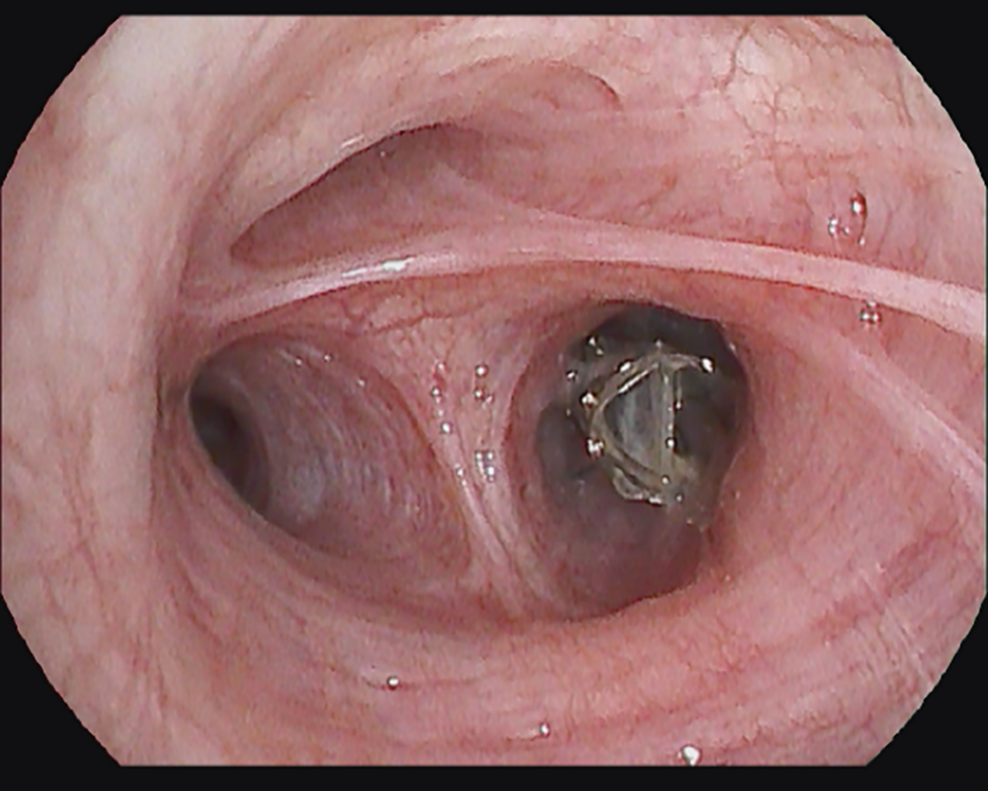
Position of the unidirectional endobronchial valve in B2 segmental branch.

Given the PAL despite standard treatments, the option of re‐surgery, including lobectomy, was discussed. However, factors such as the potential for pleural adhesions, functional loss of lung tissue, and the patient's refusal of blood products due to religious beliefs rendered this approach less feasible. Therefore a decision was made to place a one‐way endobronchial valve. Under general anaesthesia, flexible bronchoscopy was performed to first isolate the leak using the balloon catheter of the Chartis Pulmonary Assessment System (Pulmonx Inc., Redwood City, CA, USA). By inflating the balloon in the right main bronchus and subsequently the segmental branches of the right upper lobe, while assessing the reduction of air leak on the digital Thopaz, the defect was localized.^1^ A 4.0 mm EBV (Pulmonx Zephyr) was then placed successfully in the B2 segmental branch leading to an immediate resolution of the air leak (Figure 2). The chest tube was removed 2 days later, and the patient was discharged shortly thereafter. One month after valve placement, a follow‐up CT scan showed a reduction of the pleural cavity with the EBV in place (Figure 1B). During follow‐up, patient did not experience adverse events from the EBV. The valve was removed with bronchoscopy without complications 6 months after placement, and a chest CT scan after 1 year demonstrated a limited area of atelectasis and minimal pleural thickening (Figure 1C) along with normal lung function parameters (FEV1 98% (*z*‐score −0.18), FVC 98% (*z*‐score −0.15), TLC 97% (*z*‐score −0.25) DLCOcSB 117% (*z*‐score 0.96)).

## DISCUSSION

Bronchopleural fistula and parenchymal defects following pulmonary infections or resections present significant challenges, traditionally addressed through surgery.^2^ However, not all patients are suitable for surgical intervention, and repeated surgeries may offer limited clinical benefit or irreversible functional decline. In our case, a 17‐year‐old patient developed a PAL due to necrotizing pneumonia with a bronchopleural fistula and empyema. VATS was performed for source control, seeing our high suspicion of a bronchopleural fistula on imaging. Intrapleural enzyme therapy can be used addressing empyema in the absence of a bronchopleural fistula, although the presence of necrotizing pneumonia gives a higher change of therapy failure.^3^

Addressing the PAL in this case, re‐surgery was considered, but endobronchial intervention was preferred to preserve lung tissue, especially given the patient's young age. Unlike irreversible options such as cyanoacrylate glue, coils, or spigots,^4^ one‐way endobronchial valves can be easily removed after fulfilling their purpose, making them the least invasive option.^2^, ^5^ Although originally designed for bronchoscopic lung‐volume reduction in patients with advanced emphysema, several case series have shown their use in treatment of PAL, whether due to infection or surgery.^2^, ^5^ However, their application in children has been less common. Our case shows how viable this option can be in a young population to preserve lung tissue and how impressive the plasticity of the lung can be.

In conclusion, this case, demonstrating minimal loss of lung tissue in a necrotizing pneumonia case with bronchopleural fistula with persistent air leaks post‐surgery, suggests that one way endobronchial valve placement is a promising minimally invasive procedure for managing these persistent air leaks.

## CONFLICT OF INTEREST STATEMENT

None declared.

## ETHICS STATEMENT

We declare to have a written informed patient consent for the publication of this case report.

## Data Availability

Data sharing is not applicable to this article as no new data were created or analyzed in this study.
